# Outcome of Nellix-EVAS: single center mid-term results

**DOI:** 10.1186/s42155-019-0058-0

**Published:** 2019-04-23

**Authors:** Arash Najafi, Gabriel Tobias Sheikh, Pius Wigger, Christoph A. Binkert

**Affiliations:** 1 Department of Radiology and Nuclear Medicine, Canton Hospital Winterthur, 8401 Winterthur, Switzerland; 2 Department of Vascular Surgery, Canton Hospital Winterthur, 8401 Winterthur, Switzerland

**Keywords:** Nellix, EVAS, Endoleak, Migration

## Abstract

**Background:**

Endovascular aortic sealing (EVAS) using the Nellix system was a new approach to reduce the frequency of type II endoleaks after endovascular aortic repair. We analyzed the mid-term results, specifically looking at device migration, endoleaks and subsequent necessary secondary interventions.

**Results:**

Ten patients underwent elective EVAS treatment during our study period. 7 patients were within the IFU while 3 patients had a proximal neck shorter than 10 mm. Technical success rate was 100% and there were no short-term vascular complications. One patient died from urosepsis 14 days after the procedure and was excluded from further analysis.

A total of 6 out of 9 patients (67%) experienced device complications such as proximal graft kinking, limb separation or caudal migration. 5 also showed type Ia endoleak.

**Discussion:**

While no complication occurred short-term (up to 12 months), the Nellix system showed a high percentage of limb separation, caudal graft migration, and type Ia endoleak on mid-term follow-up, likely due to insufficient proximal anchoring of the device. Possible salvage treatments are discussed.

## Background

Endovascular repair of abdominal aortic aneurysms (AAA) is popular due to its minimal-invasiveness with reported lower peri-procedural morbidity and mortality (Patel et al., 2016). Devices are developed continuously in order to improve the outcome of endovascular therapies. The standard endograft concept with proximal and distal fixation and sealing is associated with a risk of endoleak occurrence, most commonly type II, a major contributor of secondary re-interventions (United Kingdom EVAR Trial investigators et al., 2010; Powel et al., 2017).

The Nellix Endovascular Aneurysm Sealing System (EVAS; Endologix, Irvine, California, USA) was specifically developed to overcome this type of adverse effect by not just sealing the device at the top and bottom, but instead filling the aneurysm sac, thereby preventing retrograde flow into the sac via lumbar or mesenteric branches. To achieve this, the Nellix endograft consisted of two polymer-filled polyurethane EndoBags surrounding two balloon-expandable stentgrafts. In recent years, several articles have been published showing promising early results with very low rates of secondary interventions (Zerwes et al., 2016; Gossetti et al., 2018), but so far data on mid- to long-term clinical outcomes is infrequent. We would like to present follow-up data up to 46 months.

## Methods

We looked at the mid-term results of all patients that underwent EVAS at our center between March 2013 and July 2016. Data on patient age and sex, aneurysm size and shape, proximal neck length, diameter, and angulation was gathered (Table 1). Nellix graft limb length varied between 12 cm and 20 cm depending on length of the aorta.

**Table 1:** Patient data and characteristics of abdominal aorta aneurysms

Patient	Age (years)	Sex	Aneurysm Size and Shape	Proximal Neck Diameter	Proximal Neck Length and Shape	Initial Neck Angulation
1	73	M	5.8 cm; stomach-shaped	26 mm	8 mm; conal	40°
2	85	M	5.5 cm; fusiforme	30 mm	22 mm; conal	5°
3	70	M	5.8 cm; fusiforme	23 mm	4 mm; cylindrical	30°
4	75	M	4.8 cm; fusiforme	28 mm	20 mm; cylindrical	5°
5	68	M	5.2 cm; fusiforme	32 mm	45 mm; cylindrical	7°
6	76	M	4.7 cm; fusiforme	20 mm	55 mm; cylindrical	47°
7	68	M	5.6 cm; fusiforme	23 mm	18 mm; cylindrical	55°
8	83	W	5.7 cm; stomach-shaped	25 mm	6 mm; cylindrical	7°
9	79	W	5.3 cm; fusiforme	22 mm	19 mm; conal	0°
10	76	M	5.5 cm; fusiforme	31 mm	10 mm; cylindrical	22°

Post-procedural CT-angiographic follow-ups at 3, 6, 12, 24, and 36 months were retrospectively assessed for complications such as stent graft migration, limb separation, and endoleaks leading to subsequent secondary interventions. The follow-up schedule was only altered in case of complication. Follow-up periods ranged from 24 to 46 months with a mean of 34.4 months.

## Results

A total of 10 patients underwent elective EVAS treatment for abdominal aortic aneurysm during the study period. Technical success rate of the procedure was 100%. All stent grafts were placed directly below the renal arteries. According to the instructions for use (IFU) valid from 2013 to 2016, 7 patients were within the criteria while 3 patients had a proximal neck shorter than 10 mm (4 mm, 6 mm, and 8 mm; see Table 1). There were no short-term complications and patients were discharged within a few days (median: 3.5 days; mean: 4.8 days; range 2–18 days). One patient was readmitted 9 days after the procedure due to a urinary tract infection and subsequently died 5 days later from urosepsis. He was excluded from further analysis.

**Fig. 1:**
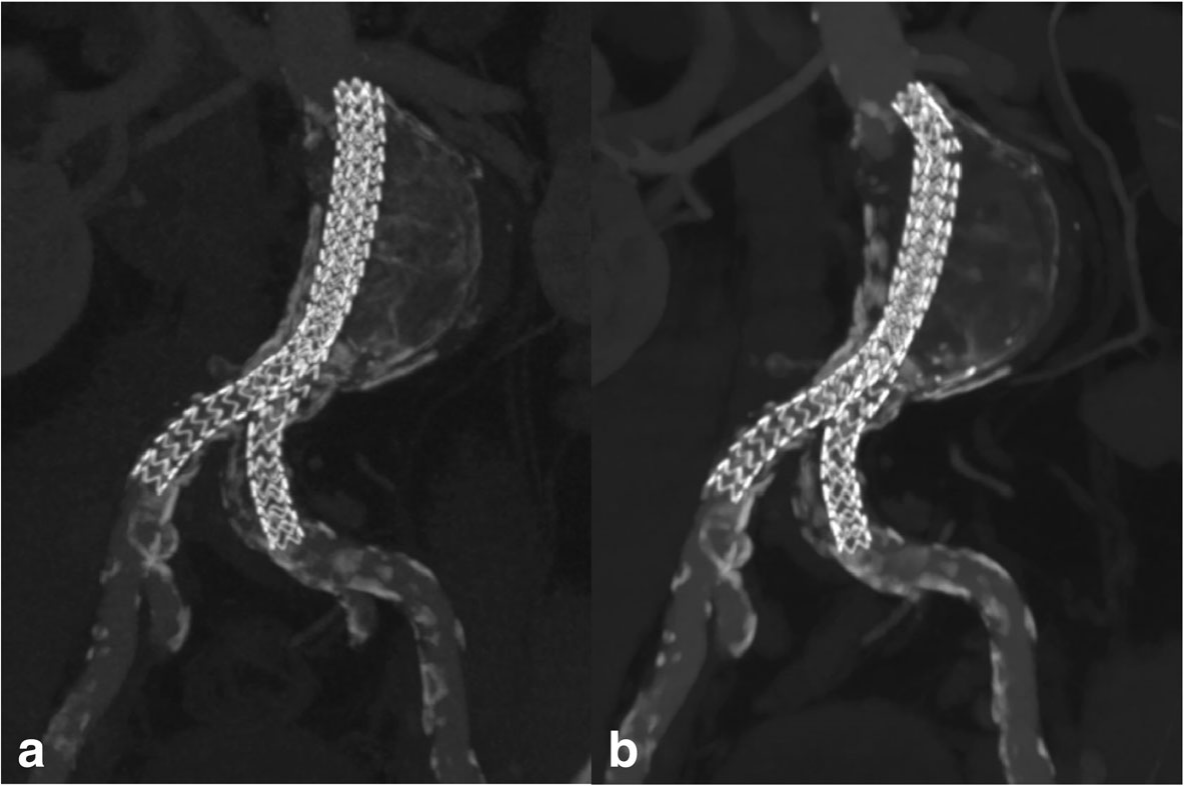
a) CT-Angio 24 months after stentgraft placement. The two graft limbs are intact and unchanged in position, conformation, and alignement. Note the stomach-shaped aneurysm sac b) CT-Angio 36 months after stentgraft placement. There is proximal kinking with slight shift of both limbs towards the “greater curvature” of the stomach-shaped aneurysm with minimal limb separation and caudal migration

**Fig. 2:**
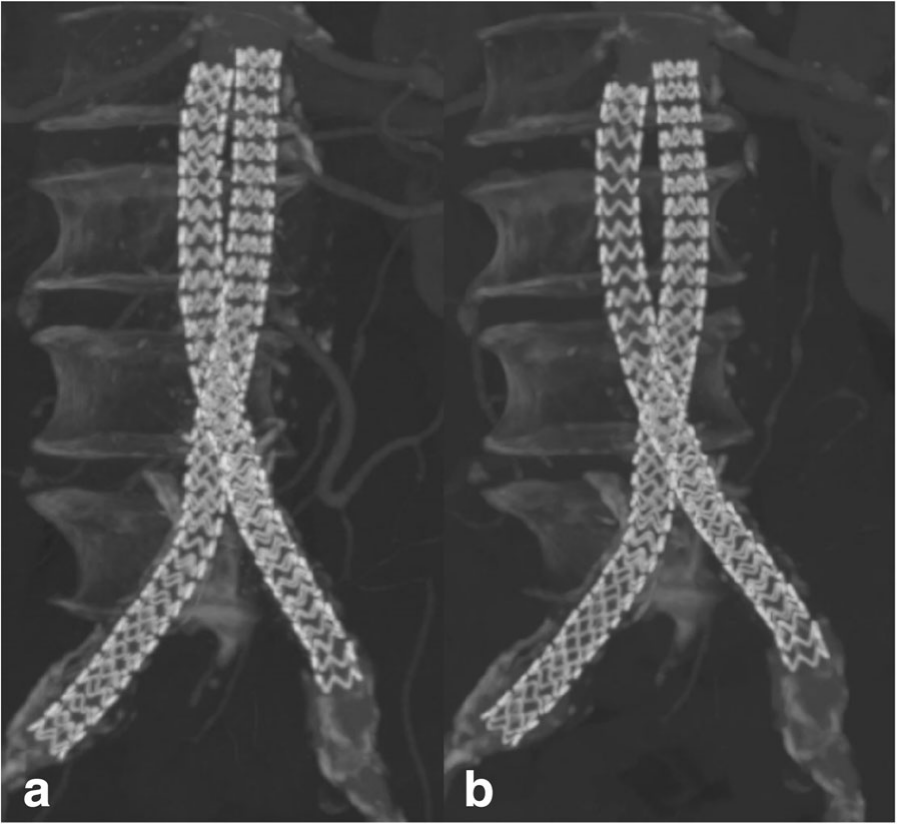
a) CT-Angio 24 months after stentgraft placement. The two graft limbs are intact and unchanged in position, conformation, and alignement. b) CT-Angio 36 months after stentgraft placement. There is conformational change of the proximal parts with separation of the limbs and minimal caudal migration

**Fig. 3:**
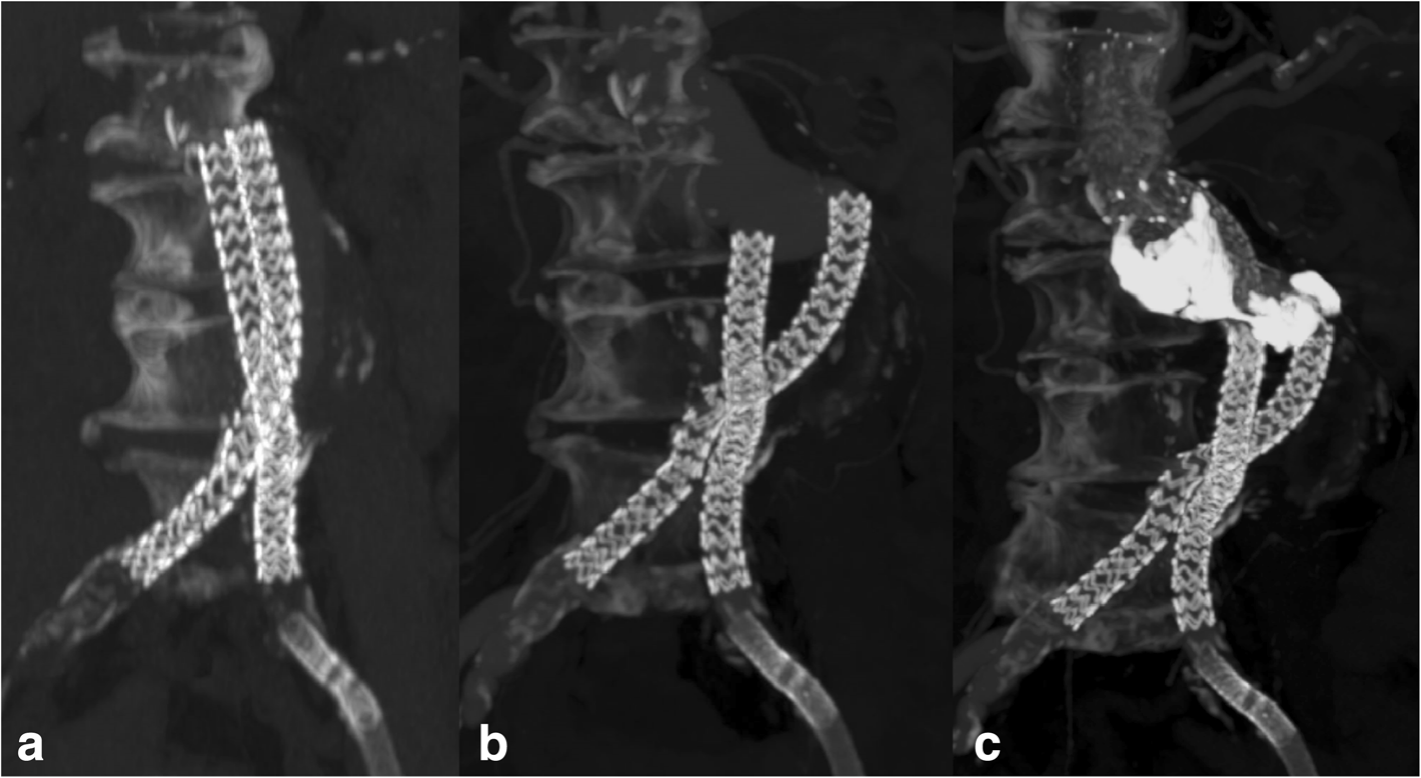
a) CT-Angio 24 months after stentgraft placement. The two graft limbs are intact and unchanged in position, conformation, and alignement. b) CT-Angio 36 months after stentgraft placement. The limbs have separated and fallen into the stomach-shaped aneurysm. Note the type Ia endoleak. c) CT-Angio 1 month after secondary intervention: 1. Extension of both limbs with stentgrafts until beneath the renal arteries 2. Deployment of additional bare stents above the renal arteries for further stabilization 3. Injection of liquiq embolics (Onyx) into the aneurysmatic sac to seal the type Ia endoleak. Renal arteries were normally perfused and the endoleak sealed after treatment

Device complications (Table 2) occurred in 6 patients (67%) and were observed either after 12 months or 36 months. They included new proximal angulation of the stentgrafts (in 2 patients; Fig. 1), separation of the limbs > 2 mm (in 6 patients, Fig. 2), caudal migration (in 6 patients; Fig. 3), and type Ia endoleak (5 patients). No type II endoleaks were recorded in our cohort.

**Table 2:** Summary of complication characteristics and salvage interventions

Patient	Time to Device Complication	Complications				Treatment
		New Limb Angulation	Limb Separation	Caudal Migration	Endoleak	
1	36 months	42°	2 mm	4 mm	Type Ia	Endovascular (proximal graft extension with chimney of left renal artery and coiling of aneurysm sac)
2	36 months	10°	3 mm	15 mm	Type Ia	Endovascular (proximal graft extension and liquid embolization of aneurysm sac)
3	12 months	ø	7 mm	15 mm	Type Ia	Initially endovascular (proximal graft extension and liquid embolization of aneurysm sac), then salvage surgery 16 months later
4	36 months	ø	4 mm	4 mm	Type Ia	Endovascular (proximal graft extension and liquid embolization of aneurysm sac)
8	36 months	ø	25 mm	48 mm	Type Ia	Endovascular (proximal graft extension and liquid embolization of aneurysm sac)
9	12 months	ø	4 mm	6 mm	ø	Endovascular (proximal graft extension)

Type Ia endoleak was detected in 5 out of 6 cases (83%) and always associated with caudal migration and separation of the two stentgraft limbs. Interestingly, graft migration and limb separation were concomitant in all cases (Figs. 1 and 2). There was no case of endoleak without migration or limb separation.

All 6 patients with caudal graft migration received secondary interventions to extend both graft limbs proximally and 5 of those patients were treated with additional embolization of the aneurysm sac to seal the gap between the two extended graft limbs. If deemed safe, Onyx was used as an embolic agent (*n* = 4), otherwise coiling (*n* = 1) was performed. One patient underwent conversion to open surgery with graft removal and surgical aortic repair after initial endovascular treatment was not successful. There is not sufficient follow up data on outcome of secondary treatment. Up to date, no device related mortality has been documented.

## Discussion

The Nellix EVAS system was a new concept that targeted the problem of type II endoleaks. Additionally, due to a required proximal neck length of only 10 mm in the original IFU, compared to 15 mm with most of the standard EVAR systems, potentially more patients could be treated using an endovascular approach.

Consistent with the current literature our technical success rate was 100% and no immediate post-interventional complication occurred.

However, there were six patients with complications during follow up, all of them with limb separation and caudal migration of the stent graft, often with concurrent type Ia endoleak. The early migrations occurred after just 12 months in patients with both short and long neck (4 mm and 19 mm), the late migrations after 36 months, again in patients with both short and long necks (6 mm, 8 mm, 20 mm, and 22 mm), indicating that neck length per se might not be the major contributing factor to migration.

The Nellix device had no hooks or other anchoring system to prevent limb separation or caudal migration. In addition, the stent grafts were balloon-expandable stents which could not adapt to any conformational changes of the aorta. Presumably due to better fixation of the distal parts of the stent grafts in the iliac arteries, migration appears to mainly affect the proximal parts of the stent grafts with caudal displacement leading to type 1a endoleaks. In fact, in the study from Dorweiler et al., (2017) there was 1 patient out of 24 that experienced deviation of the proximal limb segments. That patient was the only one with type Ia endoleak and aneurysm growth at 12 months. Hence it can be concluded that conformational changes in form of limb separation in the proximal graft segments are important indicators of an impending type Ia endoleaks while changes in the iliac segments are probably benign. Whether limb separation precedes or comes after caudal migration cannot be concluded from our data, but in our study they were always concomitant and more importantly highly associated with type Ia endoleaks.

Possible endovascular therapy options for migration and type Ia endoleaks in patients with the Nellix EVAS system include stent graft extension of both limbs with stent grafts and filling of the endoleak space either with coils or liquid embolization material. In our study 5 out of 6 patients could sufficiently be treated endovascularly, at least in the short term. In one case endovascular repair failed (progressive limb separation with persistent type 1a endoleak) and therefore open surgical aortic repair was performed as a salvage therapy.

Our study is limited by size and partial non-adherence to IFU in a third of our patients but nevertheless displays a weakness of EVAS. Even if only patients with neck lengths according to IFU were considered, the complication rate would still amount to 33%. We do not yet have short- or mid-term results of secondary intervention outcomes in all patients.

## Conclusion

The Nellix EVAS system had been proven to fulfill the objective of eliminating type II endoleaks and as presented at the Charing Cross International Symposium in April 2018, seems to have lower mid-term mortality rates than conventional EVAR systems. However, our study showed a high percentage of stent graft angulation, graft migration, limb separation and ultimately type Ia endoleak at mid-term follow-up, likely due to insufficient proximal anchoring of the device. It is important therefore to specifically look for these device-specific complications during follow-up for patients who have received these endografts. An improved proximal stent fixation could possibly help to improve the mid-term success rate of EVAS.

These device issues have been recognized by Endologix and they have released a field safety notice at the beginning of 2019, effectively ceasing sales due to the aforementioned adverse events.

## Abbreviations

AAA: Abdominal aortic aneurysms
EVAS: Endovascular Aneurysm Sealing System
IFU: Instructions for use
